# *Bartonella* spp. in Feral Pigs, Southeastern United States

**DOI:** 10.3201/eid1705.100141

**Published:** 2011-05

**Authors:** Adam W. Beard, Ricardo G. Maggi, Suzanne Kennedy-Stoskopf, Natalie A. Cherry, Mark R. Sandfoss, Christopher S. DePerno, Edward B. Breitschwerdt

**Affiliations:** Author affiliation: North Carolina State University, Raleigh, North Carolina, USA

**Keywords:** swine, pigs, Bartonella vinsonii subsp. berkhoffii, Bartonella henselae, Bartonella koehlerae, bacteremia, bacteria, zoonoses, United States, dispatch

## Abstract

In conjunction with efforts to assess pathogen exposure in feral pigs from the southeastern United States, we amplified *Bartonella henselae, B. koehlerae*, and *B. vinsonii* subsp. *berkhoffii* from blood samples. Feral pigs may represent a zoonotic risk for hunters or butchers and pose a potential threat to domesticated livestock.

*Bartonella* spp. are intravascular, gram-negative bacteria that infect a diverse array of wild and domestic animals. These bacteria appear to induce a wide range of symptoms in humans and can cause similar disease manifestations in animals (*1**,**2*). An increasing number of *Bartonella* spp. are regarded as zoonotic pathogens, which creates a public health concern for human and veterinary medicine (*3*).

Feral pigs (*Sus scrofa*), nonnative, ancestral species derived from domesticated pigs in Europe, inhabit 39 states. As their geographic distribution expands and their numbers increase, these animals are causing substantial economic and ecologic damage, which has required implementation of specific damage management programs (*4*). Hunters and butchers coming in contact with blood from feral pigs may be at risk for infection with *Bartonella* spp (*3*). We report the molecular detection of 3 zoonotic *Bartonella* spp. in feral pigs harvested by hunters in Johnston County, North Carolina, USA.

## The Study

During 2007–2009, a total of 135 EDTA-anticoagulated whole blood samples were obtained from 76 hunter-harvested juvenile and adult feral pigs (39 males). Blood samples were aspirated postmortem from the carotid artery, heart, or orbital venous sinus, resulting in >1 blood sample for 57 feral pigs. Specimens were stored frozen at –20°C until analysis.

DNA was extracted from EDTA anticoagulated whole blood with QIAGEN MagAttract DNA Blood Mini M48 Kits and QIAGEN BioRobot M48 Workstation (QIAGEN, Valencia, CA, USA) according to the manufacturer’s instructions. All 135 samples were initially screened for the *Bartonella* 16S–23S internal transcribed spacer (ITS) region by using oligonucleotides 438s (5′-GGT TTT CCG GTT TAT CCC GGA GGG C-3′) and 1100as (5′-GAA CCG ACG ACC CCC TGC TTG CAA AGC A-3′) as forward and reverse primers, respectively (*5**–**7*). Samples with positive ITS results were subsequently screened with citrate synthase, RNA polymerase B (*rpoB*), and a *B. koehlerae*–specific PCR (*6*).

For this study, a newly designed forward ITS primer (Bkoehl-1s (5′-CTT CTA AAA TAT CGC TTC TAA AAA TTG GCA TGC-3′) was used in conjunction with the 1100as reverse primer. Amplification was performed in a 25-µL final volume reaction containing 12.5 µL of Tak-Ex Premix (Fisher Scientific, Pittsburgh, PA, USA), 0.1 µL of 100 µmol/L of each forward and reverse primer (IDT DNA Technology, Coralville, IA, USA), 7.3 µL of molecular grade water, and 5 µL of DNA from each sample tested. Blood from a healthy dog was routinely used during DNA extraction and as a PCR negative (5 µL of extracted DNA) control. For positive controls, 5 µL of 0.001 pg/µL of *B. henselae* DNA (equivalent to 2.5 genome copies) was prepared by serial dilution in specific pathogen-free dog blood (*7*). No positive control was used for the *B. koehlerae* PCR. Conventional PCR was performed in an Eppendorf Mastercycler EPgradient (Eppendorf, Hamburg, Germany) under the following conditions: 1 denaturing cycle at 95°C for 2 min followed by 55 cycles at 94°C for 15 s, 68°C (*Bartonella* genus PCR) or 64°C (*B. koehlerae* PCR) for 15 s, and 72°C for 18 s. PCR was completed by an additional final cycle at 72°C for 30 sec. Products were analyzed by 2% agarose gel electrophoresis and detection by using ethidium bromide under UV light and sequenced either after purification of amplicons directly from the gel or from plasmid-clone minipreps by using QIAquick PCR purification kit or QIAGEN Miniprep Kit (QIAGEN), respectively, as described (*6**,**7*).

Sequence chromatograms and sequence analysis were examined by using ContigExpress software (Vector NTI Suite 10.1, Invitrogen Corp., Carlsbad, CA, USA) and BLAST version 2.0 (www.ncbi.nlm.nih.gov/Education/BLASTinfo/BLAST_algorithm.html) from GenBank. Bacteria species and strain identification was performed by using AlignX software (Vector NTI Suite 10.1, Invitrogen).

Of 76 feral pigs harvested from Johnston County, North Carolina, and tested by using the 438–1100 ITS PCR, amplicons consistent in size with a *Bartonella* spp. (400–600-bp amplicon size) were amplified and successfully sequenced from 15 (19.7%) animals. Two *B. henselae* strains, *B. koehlerae* and *B. vinsonii* subsp. *berkhoffii* genotypes I and III, were identified (Figure). Seven *Bartonella* PCR–positive samples aligned with *B. koehlerae* with sequence similarities of 99.2%, 99.4%, 99.8%, and 100% (4 animals) to GenBank sequence AF312490. Four sequences aligned with *B. henselae* strain Cal-1 (GenBank accession no. AF369527) with 98.7%, 99.1%, 99.4% (2 animals) sequence similarities. *B. henselae* strain SA2 (San Antonio 2, GenBank accession no. AF369529) was detected in an additional animal with sequence homology of 99.8%.

**Figure:**
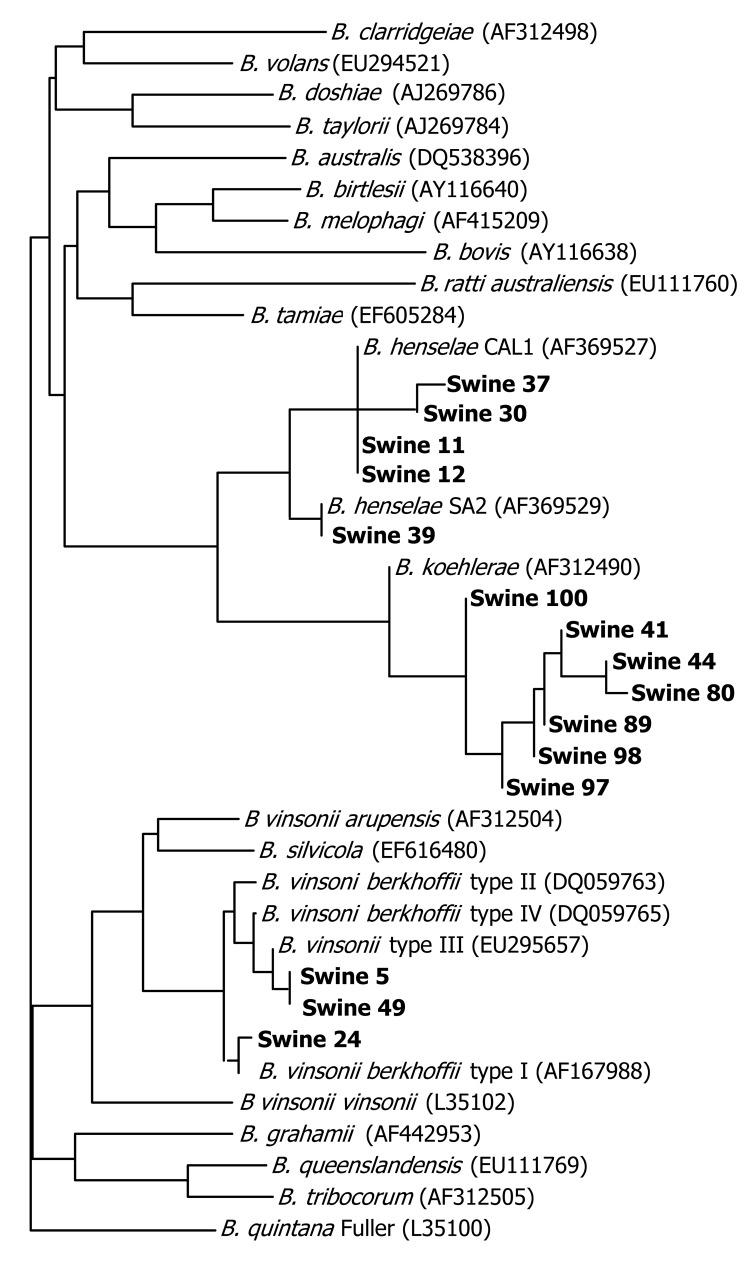
Tree pair-wise alignment of *Bartonella* DNA sequences detected in feral pig blood samples. GenBank accession numbers are in parentheses. **Boldface** indicates sequences generated in this study compared with sequences previously submitted to GenBank.

Three feral pig sequences aligned with 2 genotypes of *B. vinsonii* subsp. *berkhoffii* (*8*): 2 animals with 100% homology to genotype III (GenBank accession no. DQ059764), and 1 animal with 99.6% homology with genotype I (GenBank accession no. AF167988). *B. vinsonii* subsp. *berkhoffii* genotype III *(*99.8% homologous to DQ059764) and *B. koehlerae* (99.1%, homologous to AF312490) sequences were amplified from the same sample. Two different primer sets amplified *B. koehlerae* DNA from 3 of 7 and 1 of 2 *B. vinsonii* subsp. *berkhoffii* genotype III–infected pigs, respectively. PCR specific for the *rpoB* gene resulted in amplification of *B. vinsonii* subsp. *berkhoffii* DNA from the only *B. henselae* SA2–infected pigs. In no instance was *B. henselae* (Cal1) amplified and sequenced by using 2 primer sets. *Mesorhizobium* sequences were obtained from most of the other *rpoB* PCR amplicons and from one 325s amplicon. Previously, we have reported nonspecific amplification of *Mesorhizobium* sequences by using other *Bartonella* spp. 16S–23S ITS primers (*5*).

## Conclusions

We amplified and sequenced *B. henselae*, *B. koehlerae*, and *B. vinsonii* subsp. *berkhoffii* DNA using >1 primer sets from 19.7% of hunter-harvested feral pigs. The domestic cat is the primary reservoir for *B. henselae* and *B. koehlerae*, and fleas are the primary vector (*1*). Managers of the study site reported the presence of feral cats, but cat numbers and interactions with feral pigs were unknown. Although feral pigs in the southeastern United States are hosts for ticks that are potential *Bartonella* vectors (*9**,**10*), the pigs in this study were harvested during the winter so no ectoparasites were found.

*Mesorhizobium*, an environmental microbe, most likely introduced during sample collection under field conditions, also was amplified by using 3 primer sets. Although unlikely, ectoparasite feces or dirt containing *Bartonella* spp. could have been similarly introduced during venipuncture. For future studies in which molecular testing is anticipated, blood should be collected aseptically.

The 3 *Bartonella* spp. found in feral pigs, *B. henselae*, *B. vinsonii* subsp. *berkhoffii*, and *B. koehlerae*, are known zoonotic pathogens (*3**,**11**,**12*). Transmission of *B. alsatica*, which infects wild rabbits in Europe, has been reported in humans with endocarditis and lymphadenitis in association with butchering wild rabbits (*13*). Because hunters and butchers are exposed to large quantities of pig blood, potential exists for *Bartonella* spp. transmission through inadvertent cuts or scratches, which has occurred with other zoonotic pig pathogens, such as *Brucella suis* (*14*).

Another potential implication of these results involves the transmission of *Bartonella* spp. from feral to domesticated pigs (*15*). *Ctenocephalides felis* and *C. canis* fleas, known vectors of *B. koehlerae and B. henselae,* have been reported to infest young pigs (*10**,**12*). Measures to control ectoparasites are commonly used by large commercial pig operations, where transmission of *Bartonella* spp. is not likely to pose a production or zoonotic risk.
